# Is a History of Gestational Diabetes Associated With Long‐Term Mental Health?—Findings From the GEMS 5‐Year Follow Up Study

**DOI:** 10.1002/brb3.70666

**Published:** 2025-07-10

**Authors:** Phyllis Ohene‐Agyei, Greg D. Gamble, Debbie Samuel, Oluwatoyin Oladimeji, Qiliang Liu, Jane E. Harding, Caroline A. Crowther

**Affiliations:** ^1^ Liggins Institute University of Auckland Grafton Auckland New Zealand

**Keywords:** anxiety, depression, follow‐up studies, hyperglycemia, mental health, pregnancy induced diabetes, quality of life

## Abstract

**Introduction:**

Gestational diabetes mellitus (GDM) is associated with poor long‐term maternal metabolic health. However, there is limited evidence on the effect of GDM on later maternal mental health. We, therefore, aimed to compare mental health outcomes between women who had GDM and women who did not five years after the index pregnancy.

**Methods:**

A follow‐up study of women diagnosed with GDM using the International Association of Diabetes in Pregnancy Study Group (IADPSG) criteria matched to a random sample of women not diagnosed with GDM for maternal age, ethnicity, and BMI. Participants completed questionnaires screening for anxiety (6‐item State‐Trait Anxiety Inventory), depression (Edinburgh Postnatal Depression Scale), or health‐related quality of life (SF‐36).

**Results:**

Of the 563 participants, 233 (41.4%) were diagnosed with GDM in their index pregnancy. At follow‐up, 152/563 (27.0%) reported an increased risk of symptoms of poor mental health, and this was similar in women who were and were not diagnosed with GDM.

There was no difference between the two groups for risk of anxiety, [aRR: 0.76 (0.50, 1.22)] or depression [aRR: 1.11 (0.74, 1.66)]. However, women diagnosed with GDM reported lower mean SF‐ 36 scores for general health [aMD: −3.91 (−7.14, −0.68)] and social functioning [aMD: −3.61 (−7.05, −0.16)] than women without GDM.

**Conclusion:**

The risk of poor mental health is common five years after birth. However, GDM diagnosis was not associated with an increased risk for anxiety or depression, although GDM may affect women's perception of their later general health and social functioning.

## Graphical abstract

Poor mental health is common among mothers five years after giving birth, with around one in four women in our study self‐reporting symptoms of anxiety, depression or poor mental health related quality of life. Despite an increased risk for poor long‐term metabolic health, a previous history of gestational diabetes was not associated with an increased risk for later anxiety and depression. However, it may affect women's perception of their later general health and social functioning.

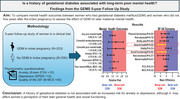

## 1 Introduction

Gestational diabetes mellitus (GDM) refers to glucose concentrations above normal but below that diagnostic of overt diabetes and first detected in pregnancy (World Health Organization 2016). According to estimates from the International Diabetes Federation, GDM affects approximately one in seven pregnant women (Wang et al. 2022), making it the most common metabolic disorder in pregnancy. In addition to short‐term complications such as gestational hypertension (Joffe et al. 1998), cesarean delivery (Tan et al. 2009), and macrosomia (Tan et al. 2009), GDM also predisposes to significant long‐term complications for mother and child.

Women with GDM have a tenfold increased risk for type 2 diabetes (Vounzoulaki et al. 2020) and a twofold increased risk for cardiovascular complications (Kramer et al. 2019) within the first 10 years after birth. Women who are diagnosed with GDM may also experience increased rates of poor mental health in the postnatal period (Arafa and Dong 2019). A meta‐analysis of 10 cohort studies reported that compared to women without GDM, women who experience GDM have a 32% increased risk for postpartum depression up to one year after birth (Arafa and Dong 2019). Importantly, maternal mental disorders in the first 15 months after birth may be associated with poor infant neurocognitive development (Koutra et al. 2013; Cornish et al. 2005) and poor mother–child interactions (McMahon et al. 2006). Children born to mothers who experience GDM have an increased risk for poor cognition and educational attainment up to 8 years after birth (Fraser et al. 2012). This suggests that in the long term, a previous diagnosis of GDM and the development of postnatal mental disorders may adversely affect mother and child health and well‐being. Thus, robust evidence is needed on risks for later poor mental health among women who experience GDM.

Currently, this evidence is limited, and the results are inconsistent (Danyliv et al. 2015; Feig et al. 1998). A cross‐sectional study in Ireland assessing self‐reported quality of life in women who had GDM 2–5 years after the affected pregnancy found that having GDM did not predispose to an increased risk for poor health‐related quality of life (HRQoL) (Danyliv et al. 2015). However, a case‐control study in Canada reported that women who had GDM reported a lower HRQoL 3–5 years after birth compared to women without a history of GDM (Feig et al. 1998).

Current evidence on the risk of anxiety and depression among women with a history of GDM is limited to the first year after birth. A retrospective cohort study in Denmark assessing the association between a history of endocrine disorders and postpartum depression reported a 50% increase in the odds of depression 6 months after birth among women who had a previous history of GDM compared to women with no such history (Rasmussen et al. 2023). Another longitudinal study in China found no association between anxiety and depression and a history of GDM one year after birth (Li et al. 2022).

To address the paucity of evidence on risk for long‐term mental disorders among women who experience GDM, this study aimed to assess the prevalence of anxiety, vulnerability to depression, and HRQoL among a multi‐ethnic cohort of women 5 years after the index birth, comparing women who were diagnosed with GDM in the index pregnancy with women who were not diagnosed with GDM.

## 2 Materials and Methods

### 2.1 Study Design and Participants

Women who participated in the Gestational Diabetes Mellitus Trial of Diagnostic Detection Thresholds (GEMS) and had not withdrawn from further follow‐up were eligible for this study. The GEMS Trial randomized participants to diagnosis of GDM using two different glycemic diagnostic thresholds—the lower International Association of Diabetes in Pregnancy Study Group (IADPSG) criteria (Metzger et al. 2008) and the higher Australasian Diabetes in Pregnancy Society (ADIPS) 2010 criteria (Hoffman et al. 1998) in use in New Zealand—to assess effects on maternal and child health and well‐being (Crowther et al. 2022).

Women who were diagnosed with GDM using the IADPSG criteria in the GEMS Trial and a randomly selected sample of women who were not diagnosed with GDM by the IADPSG criteria matched to the women who had GDM by maternal age, body mass index (BMI), and ethnicity, were invited to participate in the 5‐year study follow‐up.

Women who consented to the follow‐up study were asked to complete online questionnaires assessing health and well‐being including measures of mental well‐being. Participants who completed any of the questionnaires on anxiety, depression, or HRQoL were included in this analysis.

### 2.2 Outcome Measures

Anxiety was assessed with the 6‐item Spielberger State‐Trait Anxiety Inventory (STAI), a shortened version of the full STAI version but with similar validity and improved acceptability for research participants (Court et al. 2010). A cut‐off of >15 was assigned as an increased risk for anxiety (Crowther et al. 2005). Depression was assessed using the Edinburgh Postnatal Depression Scale (EPDS) (Cox et al. 1987) with a cut‐off of >12 used to indicate vulnerability to depression. This cut‐off has been reported to have the highest specificity in the perinatal period (Levis et al. 2020).

Mental HRQoL was assessed with the 36‐Item Short‐Form General Health Survey (SF‐36) focusing on the overall summary measure related to mental functioning (Ware and Sherbourn 1992). A cut‐off of <40 on the mental component summary (MCS), which is one standard deviation below the New Zealand population mean of 50, (Scott et al. 1999) was used to indicate poor mental health HRQoL. The eight domains of the SF‐36: general health, mental health, physical functioning, social functioning, role physical, role emotional, bodily pain, and vitality, and the overall measure relating to physical functioning were assessed as continuous scores. Higher scores on the SF‐36 domains are associated with higher levels of functioning.

### 2.3 Statistical Analysis

Statistical analyses were carried out using SAS version 9.4 (SAS Institute, Cary, North Carolina, United States of America). Baseline characteristics of all women were compared using Student's *t*‐tests or Wilcoxon's non‐parametric tests or chi‐square tests as appropriate continuous variables are reported as mean and standard deviation or median and interquartile ranges, and categorical variables as numbers and percentages. Generalized linear mixed‐effects models (GLMM) were used to generate relative risks (binomial distribution with log link function) and mean differences (normal distribution and identity link function) between the two groups. A *p* value < 0.05 was considered significant. No adjustments for multiplicity were performed. No imputation for missing data was done as the assumption that data were not missing at random was not fulfilled. The maximum likelihood estimation approach used by the GLMM statistical approach adopted does not omit data due to missingness, making it a robust approach for effectively handling missing data.

In exploratory analyses, groups were compared after stratification for the New Zealand Index of Deprivation (NZDep2018) (Atkinson et al. 2019) (less deprived, NZDep ≤ 5 vs. more deprived, NZDep > 5). NZDep2018 measures eight deprivation dimensions [communication, income (personal and household), employment, qualifications, home ownership, (family) support, living space, and living conditions] by census mesh blocks. Scoring of the index is decile‐based where 1 represents the areas with the least deprived scores and 10 the areas with the most deprived scores. The effect of subsequent pregnancy on mental health outcomes was also assessed using the Cochran‐Armitage test for trend with ordinal categories of “no subsequent pregnancy”, “subsequent pregnancy without GDM”, and “subsequent pregnancy with GDM”. Interaction analyses were performed between GDM groups and these ordinal categories using generalized linear mixed models.

## 3 Results

A total of 563 women completed questionnaires on psychological health and were included in this study (64.8% of 869 women who consented to follow‐up). Of these 233 (41.4%) were diagnosed with GDM in the index pregnancy and 330 (58.6%) were not (Table 1). The mean length of follow up was 5.4 years, although women in the GDM group completed the questionnaires approximately one year earlier than women who did not have GDM (4.8 ± 0.7years vs. 5.8 ± 0.9years). At the time of follow‐up, women who had GDM in their index pregnancy were slightly younger (37.3 ± 4.8 years vs. 38.6 ± 4.9) than those who did not, were more likely to live in the more deprived neighborhoods [56 (24.2%) vs. 51 (15.7%)], and were more likely to report a family history of diabetes [108/233 (46.3%) vs. 118/330 (35.8%)]. However, there was no difference in educational attainment between the two groups (Table 1). Eligible women who completed the follow up questionnaires were more likely to be of New Zealand European ethnicity and less socioeconomically deprived compared to those who did not (Table S1).

**TABLE 1:** Baseline characteristics of participants who had GDM in the index pregnancy compared to those who did not have GDM.

Characteristics	GDM (*N* = 233)	No GDM (*N* = 330)	*p* value
**At GEMS trial enrolment**			
Age, years (mean±SD)	32.2 ± 4.7	32.6 ± 4.8	0.38
BMI, kg/m^2^ (median, IQR) *n* (%) <25 25.0–<30 ≥30	28.2 (24.7, 32.5) 64 (27.5) 78 (33.5) 91 (39.1)	27.1 (23.7, 31.0) 109 (33.0) 118 (35.8) 103 (31.2)	0.03 0.13
Ethnicity, *n* (%) New Zealand European Māori Pacific Asian Other/MELAA	78 (33.5) 12 (5.2) 28 (12.0) 100 (42.9) 15 (6.4)	136 (41.2) 20 (6.1) 26 (7.9) 130 (39.4) 18 (5.4)	0.25
Nulliparity, *n* (%)	119 (51.1)	164 (49.7)	0.75
Previous perinatal death, *n* (%) *N* = 312	6 (5.0)	8 (4.2)	0.78
Chronic hypertension, *n* (%)	10 (4.3)	22 (6.7)	0.23
Family history of diabetes, *n* (%)	108 (46.3)	118 (35.8)	0.01
Gestational age at OGTT, weeks (median, IQR)	27.3 (26.1, 28.3)	27.1 (26.3, 28.1)	0.75
Results of OGTT, mmol/L (median, IQR) Fasting 1‐h postprandial 2‐h postprandial	4.9 (4.4, 5.3) 10.1 (9.2, 10.8) 8.6 (7.1, 9.5)	4.3 (4.2, 4.9) 7.3 (6.2, 8.1) 5.9 (5.0, 6.9)	<0.001 <0.001 <0.001
**At time of follow‐up**			
Duration of follow up, years (mean±SD)	4.8 ± 0.7	5.8 ± 0.9	<0.001
Age, years (mean±SD)	37.3 ± 4.8	38.6 ± 4.9	0.003
BMI, kg/m^2^ (median, IQR) *n* (%) <25 25.0–<30 ≥30	27.7 (23.8, 32.8) 81 (34.8) 66 (28.3) 86 (36.9)	26.3 (22.9, 31.2) 126 (38.5) 98 (30.0) 103 (31.5)	0.04 0.40
Socioeconomic deprivation (NZDep index) (Atkinson et al. 2019), *n* (%) 1–2 (least deprived) 3–4 5–6 7–8 9–10 (most deprived)	*N* = 231 34 (14.7) 44 (19.1) 51 (22.1) 46 (19.9) 56 (24.2)	*N* = 324 68 (21.0) 80 (24.7) 72 (22.2) 53 (16.4) 51 (15.7)	0.03
Highest qualification^a^, *n* (%) Level 1 (lowest) Level 2 Level 3 Level 4 (highest)	*N* = 207 11 (5.3) 36 (17.4) 130 (62.8) 30 (14.5)	*N* = 291 20 (6.9) 39 (13.4) 174 (59.8) 58 (19.9)	0.27

Abbreviations: BMI, body mass index; IQR, Interquartile range; MELAA, Middle Eastern, Latin American, African; NZDep, New Zealand Index of Deprivation (Atkinson et al. 2019); OGTT, oral glucose tolerance test; SD, standard deviation.

^a^ Levels 1: secondary school; Level 2: certificates and diplomas; Level 3: (post)graduate certificates and diplomas, bachelor's (honours) degrees; Level 4: master's degrees; doctoral degrees.

### 3.1 Prevalence of Mental Health Outcomes

Overall, the prevalence of symptoms of any of anxiety, depression, or poor mental HRQoL was 27% (152/563), and of all three mental disorders comorbidly was 5.9% (33/562). There were no significant differences between participants with a history of GDM and those without for the risk of having any of the three mental disorders [RR: 0.98 (95% CI 0.74, 1.29)] or all three mental disorders [RR: 1.18 (0.60, 2.29)] (Table 2).

**TABLE 2:** Comparison of mental health outcomes.

Mental health measure	GDM n/N (%) or mean±SD	No GDM n/N (%) or mean±SD	Relative risk or mean difference (95% CI)	*p* value
Any poor mental health	62/233 (26.6)	90/330 (27.3)	0.98 (0.74, 1.29)	0.86
Comorbid anxiety/depression	18/233 (7.7)	21/330 (6.4)	1.21 (0.66, 2.23)	0.53
Comorbid poor mental health	15/233 (6.4)	18/329 (5.5)	1.18 (0.60, 2.29)	0.63
Anxiety (STAI>15)	23/233 (9.9)	43/330 (13.0)	0.76 (0.50, 1.22)	0.22
STAI score	11.4 ± 3.3	11.5 ± 3.6	−0.10 (−0.68, 0.48)	0.74
Depression (EPDS>12)	36/233 (15.4)	46/330 (13.9)	1.11 (0.74, 1.66)	0.62
EPDS score	7.2 ± 5.0	7.1 ± 4.9	0.11 (−0.72, 0.95)	0.79
Poor mental HRQoL (MCS<40)	51/233 (21.9)	70/329 (21.3)	1.03 (0.75, 1.42)	0.86
SF‐36 scores: Physical functioning Role physical Bodily pain General health Vitality Social functioning Role emotional Mental health PCS MCS	87.2 ± 21.2 88.6 ± 27.6 76.7 ± 22.5 66.0 ± 20.1 55.0 ± 14.6 80.3 ± 21.0 85.4 ± 30.0 65.3 ± 12.8 52.8 ± 7.9 44.5 ± 8.3	88.2 ± 19.7 88.0 ± 26.9 78.6 ± 20.0 69.9 ± 18.6 53.4 ± 14.7 83.9 ± 20.1 88.1 ± 27.5 65.8 ± 12.6 53.4 ± 6.9 45.0 ± 8.2	−1.11 (−4.51, 2.28) 0.74 (−3.84, 5.32) −1.92 (−5.46, 1.62) −3.91 (−7.14, −0.68) 1.50 (−0.96, 3.96) −3.61 (−7.05, −0.16) −2.67 (−7.47, 2.12) −0.45 (−2.58, 1.67) −0.63 (−1.85, 0.60) −0.56 (−1.94, 0.82)	0.52 0.75 0.29 0.02 0.23 0.04 0.27 0.67 0.31 0.43

Abbreviations: EPDS, Edinburgh Postnatal Depression Scale; HRQoL, health‐related quality of life; MCS, mental component score; PCS, physical component score; SF‐36, 36‐Item Short‐Form General Health Survey; STAI, Spielberger State‐Trait Anxiety Inventory.

Women who had GDM in their index pregnancy had a slightly lower prevalence of anxiety symptoms compared to women who did not (9.9% vs. 13.0%), but this difference was not statistically significant [RR: 0.76 (0.50, 1.22)]. Comparing the domains of the SF‐36, women who had GDM reported lower mean scores (implying worse functioning) for general health [aMD: −3.91 (−7.14, −0.68)] and social functioning [aMD: −3.61 (−7.05, −0.16)] compared to women who did not have GDM. Mean EPDS and STAI scores and the remaining domains of the SF‐36, including the two overall measures, were not significantly different between groups.

### 3.2 Exploratory Analyses

Exploratory analyses showed that among women with GDM, but not those without GDM in the index pregnancy, there was a trend for vulnerability to depression to decrease when comparing the categories of no subsequent pregnancy, subsequent pregnancy without GDM, and subsequent pregnancy with GDM (19.0% vs. 11.6% vs. 5.7%; *p* trend = 0.04), but the interaction was not statistically significant (interaction *p* value = 0.08) (Table 3). There were no significant interactions between other measures of mental health and categories of subsequent pregnancy. Interaction analyses to explore the effect of parity, BMI, and chronic hypertension showed no interaction with GDM status.

**TABLE 3:** Comparison of effect of subsequent pregnancy and recurrent GDM on mental health outcomes between the two groups.

		No subsequent pregnancy	Subsequent pregnancy without GDM	Subsequent pregnancy with GDM	*p* value for trend	interaction *p* value
Anxiety (STAI>15)	GDM (*N* = 233)	18/153 (11.8)	5/43 (11.6)	0/35 (0.0)	0.06	0.14
	No GDM (*N* = 330)	26/199 (13.1)	16/116 (13.8)	1/11 (9.1)	0.95	
Depression (EPDS>12)	GDM	29/153 (19.0)	5/43 (11.6)	2/35 (5.7)	0.04	0.08
	No GDM	31/199 (15.6)	14/116 (12.1)	1/11 (9.1)	0.32	
Poor mental HRQoL (MCS<40)	GDM	35/153 (22.9)	11/43 (25.6)	5/35 (14.3)	0.40	0.49
	No GDM	43/199 (21.6)	25/116 (21.5)	2/11 (18.2)	0.88	

EPDS, Edinburgh Postnatal Depression Scale; HRQoL, health‐related quality of life; MCS, mental component score; STAI, Spielberger State‐Trait Anxiety Inventory.

Stratified analyses assessed the interaction between GDM status and deprivation on the prevalence of mental health outcomes. In the more deprived group (NZDep2018>5), the direction of effect was for women with GDM in the index pregnancy to be less likely than those without to have any self‐reported symptoms of poor mental health [26.2% vs. 34.6%; RR: 0.76 (0.52, 1.10)], whereas in the less deprived group (NZDep2018 ≤ 5) women with GDM in the index pregnancy were more likely than those without to have self‐reported symptoms of poor mental health outcomes [27.1% vs. 22.3%; RR: 1.21 (0.81, 1.82)], but these differences and the interaction effect were not statistically different (interaction *p* value = 0.09) (Table 4). This direction of effect was seen across all the mental health outcomes assessed.

**TABLE 4:** Comparison of prevalence of mental health outcomes between groups with different levels of deprivation.

	More deprived (NZDep2018 > 5) *N* = 259				Less deprived (NZDep2018 ≤ 5) *N* = 305				
	GDM *n* (%) or mean±SD	No GDM *n* (%) or mean±SD	Relative risk or mean difference (95% CI)	*p* value	GDM *n* (%) or mean±SD	No GDM *n* (%) or mean±SD	Relative risk or mean difference (95% CI)	*p* value	Interaction *p* value
Any poor mental health	33/126 (26.2)	46/133 (34.6)	0.76 (0.52, 1.10)	0.15	29/107 (27.1)	44/197 (22.3)	1.21 (0.81, 1.82)	0.35	0.09
Comorbid anxiety and depression	10/126 (7.9)	14/133 (10.5)	0.75 (0.35, 1.64)	0.47	8/107 (7.5)	7/197 (3.6)	2.10 (0.78, 5.67)	0.14	0.11
Comorbid poor mental health	8/126 (6.3)	11/132 (8.3)	0.76 (0.32, 1.84)	0.54	7/107 (6.5)	7/197 (3.6)	1.84 (0.66, 5.13)	0.24	0.20
Anxiety (STAI>15)	12/126 (9.5)	24/133 (18.0)	0.53 (0.27, 1.01)	0.05	11/107 (10.3)	19/197 (9.6)	1.07 (0.53, 2.16)	0.86	0.15
STAI score	11.8 ± 3.3	12.2 ± 3.7	−0.37 (−1.23, 0.49)	0.39	11.0 ± 3.3	11.1 ± 3.4	−0.09 (−0.90, 0.71)	0.82	0.64
Depression (EPDS>12)	22/126 (17.5)	30/133 (22.6)	0.77 (0.47, 1.27)	0.31	14/107 (13.1)	16/197 (8.1)	1.61 (0.82, 3.18)	0.17	0.09
EPDS score	7.7 ± 4.9	8.3 ± 5.4	−0.60 (−1.86, 0.67)	0.35	6.7 ± 5.0	6.3 ± 4.5	0.37 (−0.73, 1.48)	0.50	0.25
Poor mental HRQoL (MCS<40)	325/126 (19.8)	32/132 (24.2)	0.82 (0.51, 1.30)	0.40	26/107 (24.3)	38/198 (19.2)	1.27 (0.81, 1.97)	0.29	0.18
SF‐36 scores: Physical functioning Role physical Bodily pain General health Vitality Social functioning Role emotional Mental health PCS MCS	83.2 ± 24.3 85.6 ± 31.1 74.8 ± 24.4 66.2 ± 19.6 54.4 ± 14.9 77.6 ± 21.7 85.2 ± 29.7 64.0 ± 13.0 51.5 ± 8.5 44.4 ± 8.0	86.5 ± 21.9 87.3 ± 28.1 77.2 ± 21.4 68.8 ± 18.2 53.9 ± 15.1 81.7 ± 20.6 89.7 ± 27.0 63.9 ± 13.3 53.0 ± 6.6 44.7 ± 7.9	−3.21 (−8.86, 2.45) −1.69 (−8.93, 5.54) −2.47 (−8.08, 3.14) −3.54 (−8.18, 1.09) 0.49 (−3.17, 4.16) −4.09 (−9.26, 1.08) −4.54 (−11.47, 2.39) 0.63 (−2.59, 3.85) −1.50 (−3.36, 0.36) −0.28 (−2.23, 1.67)	0.26 0.64 0.39 0.13 0.79 0.12 0.20 0.70 0.11 0.78	91.9 ± 15.6 92.1 ± 22.4 79.0 ± 20.0 66.9 ± 20.6 55.6 ± 14.3 83.4 ± 19.7 85.7 ± 30.4 66.3 ± 12.5 54.4 ± 6.7 44.5 ± 8.7	89.6 ± 17.6 88.2 ± 26.4 79.6 ± 18.9 70.7 ± 18.8 53.2 ± 14.4 85.3 ± 19.7 87.0 ± 27.9 67.1 ± 11.9 53.8 ± 7.0 45.2 ± 8.3	2.55 (−1.51, 6.62) 3.68 (−2.21, 9.57) −0.81 (−5.36, 3.73) −3.78 (−8.37, 0.81) 2.45 (−0.94, 5.84) −1.96 (−6.59, 2.67) −1.50 (−8.25, 5.26) −0.77 (−3.63, 2.09) 0.60 (−1.04, 2.23) −0.72 (−2.71, 1.28)	0.22 0.22 0.72 0.11 0.16 0.40 0.66 0.60 0.47 0.48	0.10 0.25 0.65 0.94 0.44 0.54 0.54 0.52 0.09 0.76

Abbreviations: EPDS, Edinburgh Postnatal Depression Scale; HRQoL, health‐related quality of life; MCS, mental component score; NZDep, New Zealand Index of Deprivation (Atkinson et al. 2019); STAI, Spielberger State‐Trait Anxiety Inventory.

## 4 Discussion

In this five‐year follow‐up study, we found no differences in the risk for elevated symptoms of anxiety or depression comparing women who had GDM in their index pregnancy to those who did not have GDM. However, women who had GDM in the index pregnancy reported lower mean scores in their general health and social functioning domains of the SF‐36 (indicating worse functioning) compared to women who did not have GDM.

GDM has been identified as a risk factor for persistent depression up to 3 years postpartum (Putnick et al. 2020). Among women who receive a diagnosis of GDM, persisting poor mental health status has been related to a poor perception of well‐being during pregnancy which may continue long‐term after the birth (Feig et al. 1998). Our finding of no difference in the prevalence of elevated symptoms for poor mental health between women who had GDM and those who did not suggest that the diagnosis of GDM did not have a persistent adverse effect on participants’ mental well‐being five years after their diagnosis. This is consistent with a study in Ireland which found no difference in HRQoL two to five years after a diagnosis of GDM and the authors hypothesized that this could be due to appropriate GDM management which helps to reassure women (Danyliv et al. 2015).

However, we found women with GDM reported lower mean scores for the general health and social functioning domains of the SF‐36. Lower scores on the general health domain reflect a perception of poor personal health while lower scores on the social functioning domain indicate frequent interference with normal social activities due to physical or emotional problems (Ware and Gandek 1998). Our finding is consistent with a Canadian study that suggested that women who receive a GDM diagnosis may perceive themselves at high risk for poor later health which may translate into poor general health perceptions and limited social functioning (Feig et al. 1998). Another study in Italy also reported a perception of worse general health among women who experienced GDM compared to healthy controls 8 weeks after the birth, (Dalfrà et al. 2012) in line with our study findings at 5 years.

Compared to the mean scores for women of similar age in New Zealand, women with GDM in our study had slightly lower mean scores for both general health and social functioning domains (Scott et al. 1999). A possible reason for this could relate to the factors that have been reported to affect the perceived health and social functioning of women with GDM which differ from those in the general population. In developing a GDM‐specific HRQoL questionnaire, Mokhlesi et al. reported that concerns about GDM complications and perceived constraints about diet and lifestyle choices (including social constraints) negatively influenced the quality of life of women with GDM (Mokhlesi et al. 2019). Thus, in addition to appropriate care to improve metabolic health, long‐term follow‐up care for women with GDM should include relevant psychosocial support. We did not collect data on social support and the presence of comorbidities, factors which have been reported to significantly influence perceptions of health and functioning among women with GDM, (Kayyal et al. 2025; Marchetti et al. 2017) so we could not further explore these associations.

Exploratory analyses revealed that a subsequent pregnancy did not alter the risk for elevated symptoms of poor mental health when comparing women who had GDM to those who did not. However, we found a lower prevalence of vulnerability to depression among women with GDM who had a subsequent pregnancy and also a subsequent pregnancy with GDM. Among women with GDM, increasing parity has been associated with a higher prevalence of depressive symptoms during pregnancy, (Damé et al. 2017) but not in the postnatal period (Ohene‐Agyei et al. 2023). Our findings suggest that women may have adopted coping skills due to the previous diagnosis of GDM resulting in a better later mental health. A similar finding was reported among healthy mothers, where women with no subsequent pregnancies had higher odds for depressive symptoms at 4 years follow‐up than women who had one or more subsequent births (Woolhouse et al. 2015). In our study, although there was a similar pattern of lower prevalence of vulnerability to depression among women with subsequent births who had no GDM in their index pregnancy, this trend did not reach statistical significance.

Indicators of socioeconomic deprivation such as low maternal education level, (Putnick et al. 2020; Giallo et al. 2014) and low income (Matijasevich et al. 2015) have been associated with persisting depressive symptoms among mothers three to seven years after birth. Similarly, among women with GDM, deprivation has been associated with an increased risk for depression beyond the first year after the birth (Vounzoulaki et al. 2024). This suggests that GDM diagnosis in the context of socioeconomic deprivation may predispose to an increased risk for poor later mental health. However, stratified analysis based on deprivation in our study cohort did not show any differences in risk for mental health outcomes at follow‐up between women with GDM and those without. The New Zealand Index of Deprivation (Atkinson et al. 2019) which was used in assessing deprivation has been reported to not fully account for the confounding effect of socioeconomic status based on its neighborhood‐based methodology (Blakely et al. 2004). Hence it may not reflect individual level of deprivation in this study.

In this cohort, one in four study participants reported elevated symptoms of poor mental health at five years follow‐up. Two systematic reviews on the global prevalence of postpartum depression and anxiety noted that very few studies assessed depression and anxiety beyond 12 months after birth (Shorey et al. 2018; Goodman et al. 2016). This is a significant evidence gap as some longitudinal studies have shown that poor mental health may persist beyond the first year after birth (Putnick et al. 2020; Woolhouse et al. 2015). In a longitudinal study in Australia, 14.5% of healthy mothers reported depressive symptoms (EPDS≥13) at four years postpartum, a higher prevalence estimate than those within the first year after birth (8.1% at 3 months and 10.1% at 6 months) (Woolhouse et al. 2015). Our study estimate of 14.6% of women being vulnerable to depression at five years postpartum is similarly higher than that reported at 6 months postpartum (9.1%) using data from the GEMS Trial (Ohene‐Agyei et al. 2025).

This study has several strengths. Our findings provide new evidence on the later mental health and well‐being of women who were diagnosed with GDM using the widely recommended IADPSG criteria (Metzger et al. 2008; World Health Organization 2014). The prospective nature of the data collection among an ethnically diverse study population makes our results less prone to recall bias and generalizable to the New Zealand population. We also explored how subsequent birth and socioeconomic deprivation may affect maternal long‐term mental health.

Our study, however, has some limitations. Lack of data on important predictors of postnatal mental health status such as social support and history of mental disorders (Giallo et al. 2014) may have confounded our results. Similarly, the lack of data on lifestyle measures including diet and physical activity which affects cardiometabolic health and subsequent development of diabetes is a limitation. We also used self‐reported measures to assess mental health outcomes, which although widely used in research, are not considered diagnostic gold standards. Another limitation of our study relates to selection bias as non‐study participants were more likely to be of non‐European ethnicity and in the most deprived socioeconomic category compared to study participants, factors which have been associated with an increased risk for poor mental health. Our results may, thus, be an underestimation of the risk for elevated symptoms of poor mental health within the whole cohort, hence limiting the generalizability of our findings to the general population.

In addition, no adjustments were made for multiplicity in our analysis, hence, significant *p*‐values should be interpreted with caution.

## 5 Conclusion

In conclusion, symptoms of poor mental health are common five years after birth but are not different between women who were diagnosed with GDM in their index pregnancy and those who were not. However, the diagnosis of GDM may affect women's perception of their later general health and social functioning, emphasizing the importance of relevant psychological support to promote later mental health and well‐being among this population. Long‐term follow‐up care for women who experience gestational diabetes should prioritize not only metabolic health but also psychosocial well‐being.

## Author Contributions

**Phyllis Ohene‐Agyei**: conceptualization, investigation, methodology, data curation, formal analysis, writing–original draft, writing–review and editing. **Greg D. Gamble**: conceptualization, methodology, data curation, formal analysis, supervision, writing– review and editing. **Debbie Samuel**: investigation, data curation, writing–review and editing. **Oluwatoyin Oladimeji**: investigation, writing– review and editing. **Qiliang Liu**: data curation, writing–review and editing. **Jane E. Harding**: funding acquisition, conceptualization, methodology, supervision, writing–review and editing. **Caroline A. Crowther**: funding acquisition, conceptualization, methodology, supervision, writing–review and editing.

## Ethics Statement

Ethical approval for the GEMS Follow‐Up Study was obtained from the New Zealand Northern B Health and Disability Ethics Committee (20/NTB/297). Locality approvals were obtained from the Auckland and Counties Manukau District Health Boards and women gave written informed consent.

## Conflicts of Interest

The authors declare no conflicts of interest.

## Peer Review

The peer review history for this article is available at https://publons.com/publon/10.1002/brb3.70666.

## Supporting information




**Supplementary Table**: brb370666‐sup‐0001‐Table.docx

## Data Availability

The dataset generated during the current study is not publicly available as ethical approval for this study did not include sharing of individual data.
