# Supplementary material for: Is a History of Gestational Diabetes Associated With Long‐Term Mental Health?—Findings From the GEMS 5‐Year Follow Up Study
Source: Brain Behav. 2025 Jul 10;15(7):e70666. doi: 10.1002/brb3.70666 (PMC12242691; doi:10.1002/brb3.70666)
Supplement: Supplementary file 1 — Supplementary Table: brb370666‐sup‐0001‐Table.docx [file BRB3-15-e70666-s001.docx]

**SUPPLEMENTARY TABLE**

Supplementary Table 1: Baseline characteristics of study participants compared to non-participants

| Characteristics | Study participants (N=563) | Non-participants (N=172) | p-value |
| --- | --- | --- | --- |
| **At trial enrolment** | | | |
| Age, years (mean±SD) | 32.4±4.7 | 31.8±5.2 | 0.13 |
| BMI, kg/m^2^ (median, IQR)  n (%)  <25  25.0 - <30  ≥30 | 27.4 (24.1, 31.6)  173 (30.7)  196 (34.8)  194 (34.5) | 28.5 (25.3, 33.5)  35 (20.4)  74 (43.0)  63 (36.6) | 0.002  0.02 |
| Ethnicity, n (%)  New Zealand European  Māori  Pacific  Asian  Other/MELAA | 214 (38.0)  32 (5.7)  54 (9.6)  230 (40.9)  33 (5.9) | 39 (22.7)  11 (6.4)  34 (19.8)  80 (46.5)  8 (4.6) | <0.001 |
| Nulliparity, n (%) | 283 (50.3) | 81 (47.1) | 0.47 |
| Previous perinatal death, n (%) | 14 (4.5) | 3 (3.1) | 0.56 |
| Chronic hypertension, n (%) | 32 (5.7) | 12 (7.0) | 0.53 |
| Family history of diabetes, n (%) | 226 (40.1) | 75 (43.6) | 0.42 |
| Gestational age at OGTT, weeks (median, IQR) | 27.1 (26.3, 28.1) | 27.9 (26.6, 29.1) | <0.001 |
| Results of OGTT, mmol/L (median, IQR)  Fasting  1-hour postprandial  2-hour postprandial | 4.5 (4.2, 4.9)  8.1 (7.0, 9.7)  6.7 (5.5, 8.2) | 4.6 (4.3, 5.0)  8.0 (7.2, 9.6)  6.6 (5.7, 8.3) | 0.06  0.84  0.87 |
| **At follow-up** | | | |
| Duration of follow up, years (mean±SD) | 5.4±1.0 | 6.0±1.0 | <0.001 |
| Age, years (mean±SD) | 38.0±4.9 | 37.9±5.2 | 0.81 |
| BMI, kg/m^2^ (median, IQR)  n (%)  <25  25.0 - <30  ≥30 | 26.8 (23.3, 31.9)  207 (37.0)  164 (29.3)  189 (33.8) | 28.2 (24.8, 35.2)  43 (27.7)  48 (31.0)  64 (41.3) | 0.01  0.08 |
| Socioeconomic deprivation (NZDep index), n (%)  1-2 (least deprived)  3-4  5-6  7-8  9-10 (most deprived) | N=555  102 (18.4)  124 (22.3)  123 (22.2)  99 (17.8)  107 (19.3) | N=164  18 (11.0)  37 (22.6)  31 (18.9)  27 (16.5)  51 (31.1) | 0.01 |
| Highest qualification†, n (%)  Level 1 (lowest)  Level 2  Level 3  Level 4 (highest) | N=498  31 (6.2)  75 (15.1)  304 (61.0)  88 (17.7) | N=2  0  0  0  2 (100.0) | 0.10 |

BMI – body mass index; IQR – Interquartile range; MELAA – Middle Eastern, Latin American, African; NZDep – New Zealand Index of Deprivation^23^; OGTT – oral glucose tolerance test; SD – standard deviation

†Levels 1: secondary school; Level 2: certificates and diplomas; Level 3: (post)graduate certificates and diplomas, bachelor’s (honours) degrees; Level 4: master’s degrees; doctoral degrees
